# Improved Bathymetric Mapping of Coastal and Lake Environments Using Sentinel-2 and Landsat-8 Images

**DOI:** 10.3390/s19122788

**Published:** 2019-06-21

**Authors:** Ali P. Yunus, Jie Dou, Xuan Song, Ram Avtar

**Affiliations:** 1 The State Key Laboratory of Geohazard Prevention and Geoenvironment Protection (SKLGP), Chengdu University of Technology, Chengdu 610059, China; yunusp@cdut.edu.cn; 2 Civil and Environmental Engineering, Nagaoka University of Technology, 1603-1, Kami-Tomioka, Nagaoka, Niigata 940-2188, Japan; 3 Center for Spatial Information Science, The University of Tokyo, Chiba 277-8568, Japan; songxuan@csis.u-tokyo.ac.jp; 4 Faculty of Environmental Earth Science, Hokkaido University, Sapporo 060-0810, Japan; ram@ees.hokudai.ac.jp

**Keywords:** aquatic environment, remote sensing, topographic mapping, spectral reflectance, random forest

## Abstract

The bathymetry of nearshore coastal environments and lakes is constantly reworking because of the change in the patterns of energy dispersal and related sediment transport pathways. Therefore, updated and accurate bathymetric models are a crucial component in providing necessary information for scientific, managerial, and geographical studies. Recent advances in satellite technology revolutionized the acquisition of bathymetric profiles, offering new vistas in mapping. This contribution analyzed the suitability of Sentinel-2 and Landsat-8 images for bathymetric mapping of coastal and lake environments. The bathymetric algorithm was developed using an empirical approach and a random forest (RF) model based on the available high-resolution LiDAR bathymetric data for Mobile Bay, Tampa Bay, and Lake Huron regions obtained from the National Oceanic and Atmospheric Administration (NOAA) National Geophysical Data Center (NGDC). Our results demonstrate that the satellite-derived bathymetry is efficient for retrieving depths up to 10 m for coastal regions and up to 30 m for the lake environment. While using the empirical approach, the root-mean-square error (RMSE) varied between 1.99 m and 4.74 m for the three regions. The RF model, on the other hand, provided an improved bathymetric model with RMSE between 1.13 m and 1.95 m. The comparative assessment suggests that Sentinel-2 has a slight edge over Landsat-8 images while employing the empirical approach. On the other hand, the RF model shows that Landsat-8 retrieves a better bathymetric model than Sentinel-2. Our work demonstrated that the freely available Sentinel-2 and Landsat-8 imageries proved to be reliable data for acquiring updated bathymetric information for large areas in a short period.

## 1. Introduction

Aquatic environments are some of the most dynamic regions of the earth. Among the aquatic systems, the bathymetry or depth of underwater terrain is one of the most important parameters constantly being reworked and changed both in space and time. The rapid reworks in bathymetry are because of the changes in the patterns of energy dispersal and related sediment transport pathways [1]. Clarke [2] indicated that huge turbidity currents result in bedform migration within a few hours. Simons and Richardson [3] presented a positive correlation between bathymetric changes and measured stream power in fluvial systems. Sea level rise, shoreline morphology dynamics, beach nourishment, coastal erosion, and accretion are other relevant forcing factors behind bathymetric changes [4,5]. In shallower waters, updated and detailed coastal topography and bathymetry are critical for navigational purpose, pipeline constriction, exploration, defense, and research applications, as well as other management and spatial planning developmental projects [6,7]. However, due to the constant rework of bathymetry, the mapping and measuring of these alterations require a shift from static management measures to near-real-time management procedures [8].

Traditional or static methods for monitoring and measuring bathymetry rely on field surveys utilizing echo sounding and mapping using multi-beam and side-scan sonars. However, such approaches are characterized as being costly, labor-intensive, and time-consuming techniques. In single-beam echo sounders, a sound pulse from the vessel carrying the echo sounder instrument is sendt underneath and listens until the echo from the bottom is heard, thus providing depth at a single point. The water depth is then estimated by dividing the speed of sound by half of the time it takes for the echo to be heard. The multi-beam and side-scan sonars transmit multiple beams of sound, which represent the intensity and amplitude of reflected acoustic signals from the sea floor, resulting in an image of its physical reflectance and scattering characteristics. Although multi-beam echo sounding (MBES) surveys produce accurate bathymetric information of the surveyed area, this method is constrained by the spatial and temporal scale, expensive to operate, and unable to survey in shallow seas and marine protected areas [9,10]. It is estimated that, at the best resolution of MBES, more than 200 ship-years and billions of dollars would be needed to complete a swath survey of the seafloor [11]. Nevertheless, the current availability of accurate bathymetric charts from ship-based surveys is not available for the whole globe, because only a small fraction of the world’s aquatic environments is surveyed so far.

The increasing body of contemporary literature shows the potential of remotely sensed data in bathymetric studies [12,13,14,15,16]. In contrast to the traditional techniques described previously, remotely sensed data rely upon the understanding of the physical properties of the surface water, the bottom topography, and the atmosphere [17]. The principal motivation for the usage of satellites in bathymetric surveys is that their uniform and comprehensive global coverage can contribute to a better understanding of the topographic changes instantaneously and spatially. Radar altimeters abroad the spacecraft European Remote Sensing Satellite (ERS-1) and Geosat surveyed over global seas to obtain bathymetric information with high accuracy and moderate spatial resolution [18]. Dixon and Naraghi [19] summarized the principles of satellite altimeter measurements for predicting seafloor topography. The gravity anomalies estimated from geoid undulations are highly correlated with seafloor topography, and these anomalies help in mapping bathymetry with a radar altimeter. ERS-1 completed its near-global mapping of sea surface topography in 1995, which was then used to reproduce the seafloor topography for data-constrained and deeper oceans [20]. Because the radar altimeter uses gravity anomalies to correlate bathymetry, this method is mostly applicable for deep sea regions for mapping large seamounts and guyots [21]. Furthermore, the estimation of bathymetry from gravity anomalies includes several mathematical models and is, therefore, a complicated approach. On the other hand, airborne Light Detection and Ranging (LiDAR) bathymetry (ALB) is a useful technique for measuring the moderately to shallower deep coastal waters and lakes (30–50 m depth) from a low-altitude aircraft using a scanning, pulsed laser beam [22]. LiDAR offers about a 70% reduction in operating costs when compared with standard ship surveys [22]; however, it also has spatial and temporal constraints. Satellite LiDAR (e.g., Ice, Cloud, and land Elevation Satellite-ICESat) was also used to estimate water depth in clear waters with high accuracy in conjunction with spectro-radiometers and other remote-sensing data [23,24].

Multispectral remote-sensing datasets characterized by high spatial and temporal resolutions are the most frequently used method to estimate bathymetry on shallow water bodies such as coastal areas, estuaries, rivers, and lakes [7,25]. This is because they are relatively cheap, easy to process, and spatially extensive [26]. Multispectral data approximate the radiative transfer in water using an empirical approach to model reflectance and measured bathymetry via least squares regression analysis [27]. Such empirical methods rely on in situ bathymetric data and their relationship with water-leaving reflectance, with wavelengths typically in the visible spectrum, and the corresponding depth [23]. A variety of satellite sensors placed in orbit support this approach. In fact, optical satellite-derived bathymetric techniques began in the 1970s with the introduction of the Landsat series [12]. Since then, several studies used higher-spatial-resolution images to measure the water depth, for instance, Landsat-4 [28], Ikonos [13], Landsat-5 and Landsat-7 [29], Quickbird [30], Worldview 2 [14], Landsat-8 [7], Sentinel-2 [16], and RapidEye [5]. In heterogeneous complex water bodies, linear relationships, log-linear relationships, and band ratios may not be correctly fitted [31]. Hyper-spectral sensors that carry several narrow-wavelength bands hold the promise of providing accurate depth retrieval [32,33]; however, the number of satellites with hyper-spectral characteristics is limited. 

Recently, machine learning (ML) techniques gained popularity for deriving depth information from satellite sensors, which typically involves significant amounts of data and dealing in more complex environments. Studies involving artificial neural network [34], support vector machine [35,36], and random forest [37] models attempted to improve the performance of bathymetric retrieval algorithms in heterogeneous environments where the empirical approach was ineffective. These data-driven models are considered to be more flexible and accurate for relating satellite images to water depth data [38].

This paper utilizes Sentinel-2 and Landsat-8 images in conjunction with existing bathymetric maps for developing a satellite-derived bathymetric (SDB) algorithm. Our hypothesis involved investigating the suitability of multi-band satellite imagery as an effective tool for updating the water column depth continually through time in both lake and coastal environments. For this purpose, we attempted both empirical and machine learning models that relate depth information to satellite reflectance in the areas of interest. Because bathymetry is constantly reworking, updating information is key for safe navigation. Sentinel-2 and Landsat-8 images were selected due to their easy availability and because of their high enough temporal resolution for mapping changes in underwater topography, allowing for bathymetric retrieval without much complex pre-processing.

## 2. Materials and Methods

### 2.1. Study Area

**Figure 1:**
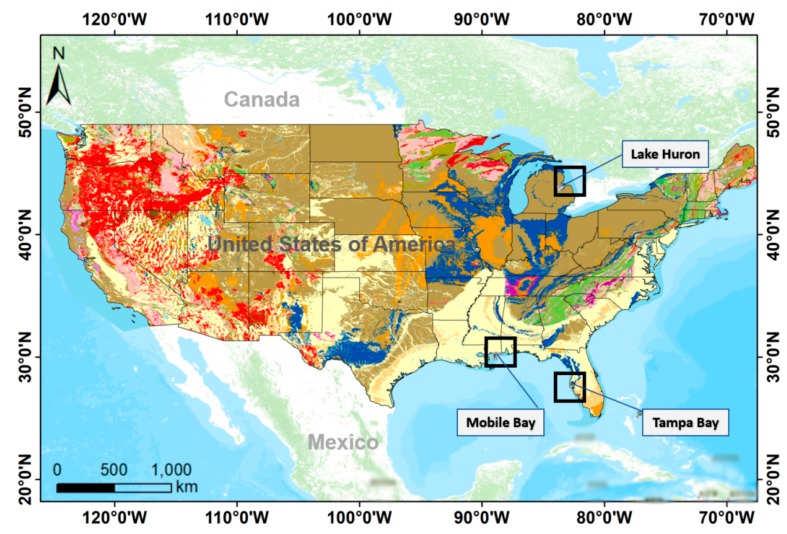
Location map of study area showing (black square boxes) Mobile Bay, Mississippi, the area adjoining Tampa Bay, and Lake Huron.

The United States (US) provides an excellent case study for the development of a systematic bathymetric retrieval approach. The governmental policy of full and open sharing, availability of high-resolution datasets, and archiving services allow researchers to initiate projects with confidence. Based on the availability of high-resolution bathymetric datasets, we chose three study areas in the conterminous United States: (i) Mobile Bay, Mississippi; (ii) the area adjoining Tamba Bay; and (iii) Lake Huron (Figure 1).

### 2.2. Dataset

#### 2.2.1. Satellite Multispectral Images 

The study takes advantage of the Sentinel-2 Multi-Spectral Imager (MSI) (hereafter referred to as Sentinel-2 or S2) and Landsat-8 Operational Land Imager (OLI) (hereafter referred as Landsat-8 or L8), which currently provide the best freely available multispectral datasets. Sentinel-2 was launched in June 2015 from French Guiana as part of the European Copernicus program, designed by Airbus Defence and Space for the European Space Agency (ESA). This sensor has 13 spectral bands covering the visible, near-infrared, and shortwave infrared parts of the electromagnetic spectrum. Table 1 shows the key summary of the 13 spectral bands of S2 sensor. Launched in February 2013, Landsat-8 acted as a successor to the Landsat-5 and 7 missions. The L8 is superior to the Landsat-5 Thematic Mapper (TM) and Landsat-7 Enhanced Thematic Mapper Plus (ETM+), with incremental improvements in satellite, sensor, transmission, reception, data processing, and data distribution technologies [39]. The temporal resolution of L8 is 16 days. Compared with Landsat-7, the L8 spectral bands remain similar except for two additional bands in the blue and shortwave infrared regions of the spectrum. A summary and key features of all eleven L8 bands are presented in Table 1.

Sentinel 2 offers a high temporal resolution of five days at the equator compared to the 16 days of Landsat-8. The swath width of S2 is 290 km as compared to 185 km for Landsat-8. The geometrically corrected S2 and L8 data, available via www.earthexplorer.gov as Level-1T (L1T) top-of-atmosphere (TOA) reflectance images and Level-1C (L1C) TOA reflectance tiles in the Worldwide Reference System (WRS-2) path/row coordinate system, are used in this study. Cloud-free datasets, one each for the three study sites were downloaded for both the S2 and L8. Dates of acquisition and path/row or tile details are also presented in Table 1. 

#### 2.2.2. Bathymetric Ddata 

The Mobile Bay bathymetric dataset was developed by the National Geophysical Data Center (NGDC), an office of the National Oceanic and Atmospheric Administration (NOAA), in March 2007. Bathymetric datasets used in the compilation of Mobile Bay include 48 National Ocean Service (NOS) hydrographic surveys, 25 US Army Corps of Engineers (USACE) surveys of dredged shipping channels, and Office of Coast Survey electronic navigational chart extracted soundings in the Chandeleur Sound region. The Tampa Bay bathymetric digital elevation model (DEM) compilation was the result of extensive collaboration between NOAA, the US Geological Survey (USGS), and other agencies such as federal and private companies. The bathymetry of Lake Huron was compiled as a component of an NOAA project to rescue Great Lakes floor geological and geophysical data. The bathymetric data for Huron were collected from USACE, the NOAA NOS, and the Canadian Hydrographic Service. These three bathymetric models were downloaded from NOAA’s National Geophysical Data Center (NGDC) at http://www.ngdc.noaa.gov/. Each of them was then resampled (from their native 3–65-m resolution) to 10 and 30 m to match with Sentinel-2 and Landsat-8 native resolutions.

### 2.3. Empirically Derived Water Depth

The bathymetry retrieval from optical satellite remote sensing is based on the wavelength-dependent attenuation of light in the water column [7]. It is known that shallow water absorbs less energy than deep water and, therefore, will have higher reflectance of solar radiation and vice versa [40]. Furthermore, in shallower waters, solar radiation is reflected back to the surface after touching the bottom depth. This water-leaving reflectance (Rrs) measured by the satellite sensor is then used to transform it into water depth via analytical equations. However, this assumption is expected to be valid only in shallow clear waters, because Rrs depends not only on the reflectance from the bottom surface, but also on the absorption and scattering properties of dissolved and suspended material in the water column. The Rrs from different spectral wavelength bands of multispectral sensors (e.g., Landsat series, Advanced Space Borne Thermal Emission and Reflection Radiometer-ASTER, RapidEye, and QuickBird) were implemented in previous studies to map bathymetry [7,10,13,40]. The blue and green wavelengths are considered to be very suitable in estimating bathymetry because reflectance between 400 and 600 nm has the deepest penetration through the water column [10]. 

**Figure 2:**
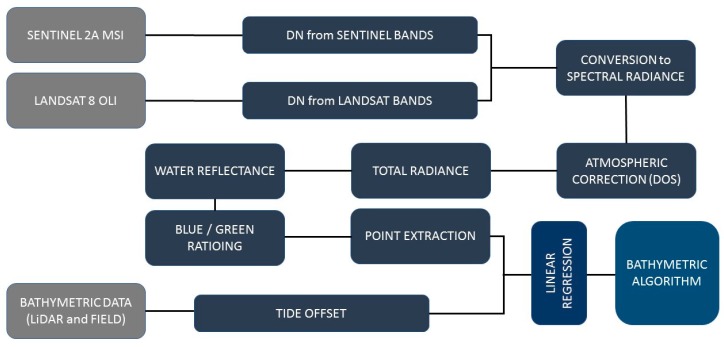
Flowchart for deriving bathymetric maps from Sentinel-2 and Landsat-8 images using empirical models.

In this study, we utilized the digital number (DN) values of band 2 (blue) and band 3 (green) of both Sentinel-2 and Landsat-8 products (Sentinel: band 2 = 490 nm and band 3 = 560 nm; Landsat: band 2 = 482 nm and band 3 = 561 nm). For this, the top-of-atmosphere (ToA) S2 and L8 products were firstly atmospherically corrected for the effects of atmospheric gases and aerosols to yield surface spectral reflectance using the dark object subtraction (DOS) method. ENVI v.5.3 software was used for pre-processing the images and for DOS calculation. In the DOS method, we assume that the dark objects (in this study, shadows in the land area adjacent to water bodies) in an image reflect no light, and any value captured by the satellite sensor is due to atmospheric scattering [20]. The atmospheric scattering effect was then removed by subtracting the value captured in dark object pixels from every pixel in the band. The corrected surface reflectance data were then used for estimating the water depth. A flowchart of methodology adapted for SDB using the empirical approach is shown in Figure 2. 

Following Pacheco et al. [7], the band ratio of blue by green (B/G) can provide satellite-derived water depth via a linear solution of water reflectance and bathymetric depth. This spectral band-ratio method employs an empirically derived formula to relate water depths to the ratio of the reflectance of two spectral bands. The depth data points were extracted in the ArcGIS environment from bathymetric LiDAR for three different sites at precisely the same locations as where the data were retrieved by the S2 and L8 images. The points for constructing a linear model were randomly selected based on the size of the available images and bathymetric data: 6000 for Mobile Bay, 3000 for Tampa Bay, and 5000 for Lake Huron. A limitation of this comparison is the fact that the bathymetric depth datasets used are dated much older than the S2 and L8 scenes. Therefore, a perfect agreement between SDB and surveyed maps is not expected, given that morphological differences are likely to occur in a moderately energetic nearshore system comprising barrier islands and tidal inlets exposed to dynamic oceanographic conditions.

### 2.4. Random Forest Model

Random forest (RF), defined as a bunch of random trees, is an ensemble learning method suitable for regression, classification, and prediction problems [41]. The advantage of the RF model is that, unlike linear models, it can capture non-linear interactions between variables. Additionally, it is good at handling both numerical and categorical data. The RF model works in the following fashion: (i) it takes a number of sub-samples from the whole dataset; (ii) at each sub-set, RF chooses a random set of features (i.e., randomly permuted at each split); (iii) based on the random sub-set, RF estimates a decision tree; (iv) finally, it aggregates all decision trees to form a single tree (forest). While performing RF regression, it uses the mean-square error (MSE) splitting criterion to measure the quality of a split, which is equal to the variance reduction. 

We implemented the random forest model for SDB using the Weka open-source machine learning software package [42]. The accuracy of machine learning models depends on the quality of data and the selection of relevant predictor variables [37,38]. A large number of training data that cover an objective data distribution produce the best fit result. To build the RF algorithm, we used 5000 random points covering a broad spectrum of depth ranging from 0–30 m for Lake Huron, and 0–10 m for Mobile Bay and Tampa Bay. Thirty percent of the sampling points were withheld from model building to be used as a test dataset; the remaining 70% were used to build the model. 

In this study, visible–near-infrared (NIR) bands of both S2 and L8 were used as predictor variables for RF training. Additionally, we used band ratios [7], log-linear ratios [12], and log-ratios [13] as predictor variables. A value of 100 was empirically set to extract the random sampling from our training dataset. The decision tree for water depth estimation was built for each sub-dataset, and its aggregate forest was used for estimating the SDB.

### 2.5. Accuracy Assessment

The uncertainties derived from the satellite-derived bathymetry (SDB) were quantified by comparing individual SDB (_Z_SDB) and reference LiDAR or surveyed bathymetry (_Z_Ref) with large sample points using the following metrics:

(i) Root-mean-square error (RMSE) is widely used for error measurements between a set of estimates and actual values, and it is a standard measure of map accuracy [43].
  $$\mathrm{RMSE}=\frac{1}{N}\sqrt{{\left(\mathrm{ZSDB}-\mathrm{ZRef}\right)}^{2}}.$$
(ii) Mean absolute error (MAE) measures the average magnitude of errors in a set of predicted values, without considering their direction. The RMSE will always be larger than or equal to the MAE.
  $$\mathrm{MAE}=\frac{1}{N}\sum |\mathrm{ZRef}-\hat{\mathrm{ZSDB}|},$$
  where ZSDB represents the predicted values, ZRef represents the actual values, and N is the number of observations.

## 3. Results

### 3.1. Site-Specific Bathymetric Algorithm (Mobile Bay)

**Figure 3:**
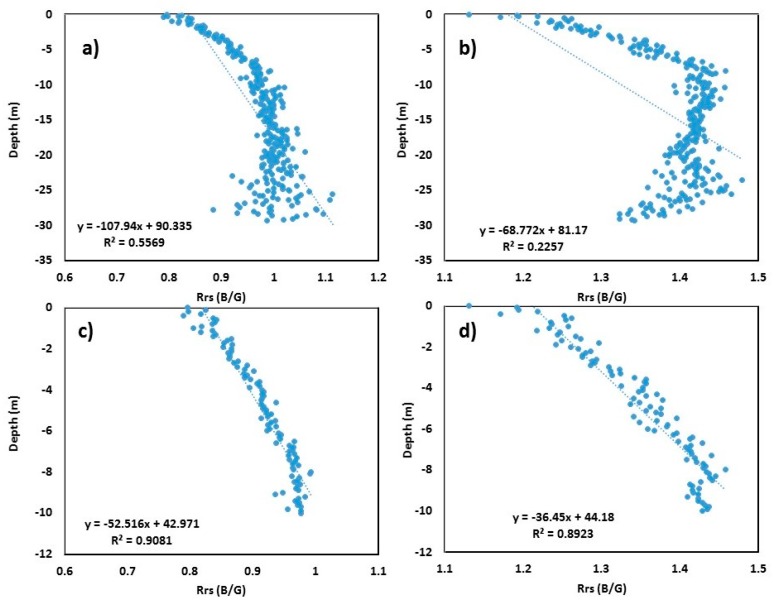
Scatter plots of blue/green (B/G) band versus surveyed depth for Mobile Bay: (**a**) Sentinel-2 B/G vs. surveyed bathymetry up to 30 m depth; (**b**) Landsat-8 B/G vs. surveyed bathymetry up to 30 m depth; (**c**) Sentinel-2 B/G vs. surveyed bathymetry up to 10 m depth; (**d**) Landsat-8 B/G vs. surveyed bathymetry up to 10 m depth.

After generating the B/G model for both S2 and L8 images, 6000 random point values were extracted from both the ratioed image and bathymetric DEM at the same place. A linear regression model was then applied to these extracted values after filtering out the land area (positive values) (Figure 3). Figure 3a,b show the ratioed image versus the surveyed bathymetric DEM linear regression model for S2 and L8, respectively, for a depth up to 30 m. The *R*^2^ values obtained were 0.55 and 0.22, respectively, for S2 and L8 images. It can be observed from the figure that, after crossing a depth of roughly 10 m, the B/G signals start scattering (Figure 3a,b). It suggests that light penetration in Mobile Bay is limited to 10 m. After adjustment of the depth up to 10 m in the linear model, the *R*^2^ values increased significantly (0.90 and 0.89 for S2 and L8, respectively; *p* < 0.001) (Figure 3c,d). This significant increase in the *R*^2^ values indicates that the bathymetry of water bodies up to the depth of 10 m could be derived from Sentinel and Landsat images.

The equation to derive depth from S2 for the Mobile Bay area is as follows:

(1) $$\mathrm{SDB}=-52.51\times \left(\mathrm{Rrs}\;\frac{B}{G}\right)+42.97.$$

The equation to derive depth from L8 for the Mobile Bay area is as follows:

(2) $$\mathrm{SDB}=-36.45\times \left(\mathrm{Rrs}\;\frac{B}{G}\right)+44.18,$$

where SDB is the satellite-derived bathymetry, and $\left(\mathrm{Rrs}\;\frac{B}{G}\right)$ is the ratio of water-leaving reflectance from the blue band to that from the green band.

### 3.2. Site-Specific Bathymetric Algorithm (Tampa Bay)

**Figure 4:**
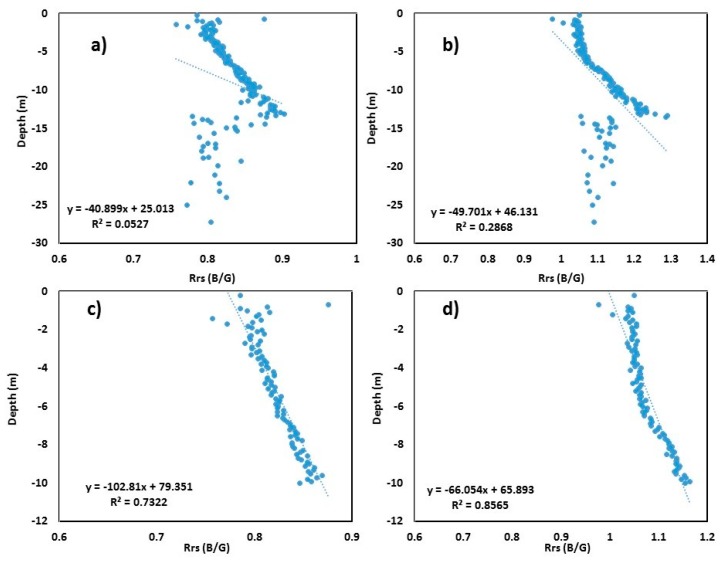
Scatter plots of blue/green (B/G) band versus surveyed depth for Tampa Bay: (**a**) Sentinel-2 B/G vs. surveyed bathymetry up to 30 m depth; (**b**) Landsat-8 B/G vs. surveyed bathymetry up to 30 m depth; (**c**) Sentinel-2 B/G vs. surveyed bathymetry up to 10 m depth; (**d**) Landsat-8 B/G vs. surveyed bathymetry up to 10 m depth.

For Tampa Bay, 3000 random point values were extracted from both the B/G image and bathymetric DEM at the same place. Linear regression was then applied to the extracted values after averaging (Figure 4). Figure 4a,b show the ratioed image versus surveyed bathymetric DEM linear regression model for S2 and L8 images, respectively, for a depth up to 30 m. The *R*^2^ values obtained were 0.05 and 0.28, respectively, for S2 and L8 sensors. Similar to Mobile Bay, it can be observed from the figure that, after crossing a depth of roughly 13 m, the B/G signals start scattering (Figure 4a,b), indicating that light penetration in Tampa Bay is limited to ~13 m. After adjustment of the depth up to 10 m in the linear model, the *R*^2^ values increased significantly (0.73 and 0.85, *p* < 0.001 for S2 and L8, respectively) (Figure 4c,d).

The equation to derive depth from Sentinel-2 for the Tampa Bay area is as follows:

(3) $$\mathrm{SDB}=-102.81\times \left(\mathrm{Rrs}\;\frac{B}{G}\right)+79.35.$$

The equation to derive depth from Landsat-8 for the Tampa Bay area is as follows:

(4) $$\mathrm{SDB}=-66.05\times \left(\mathrm{Rrs}\;\frac{B}{G}\right)+65.89.$$

### 3.3. Site-Specific Bathymetric Algorithm (Lake Huron) 

**Figure 5:**
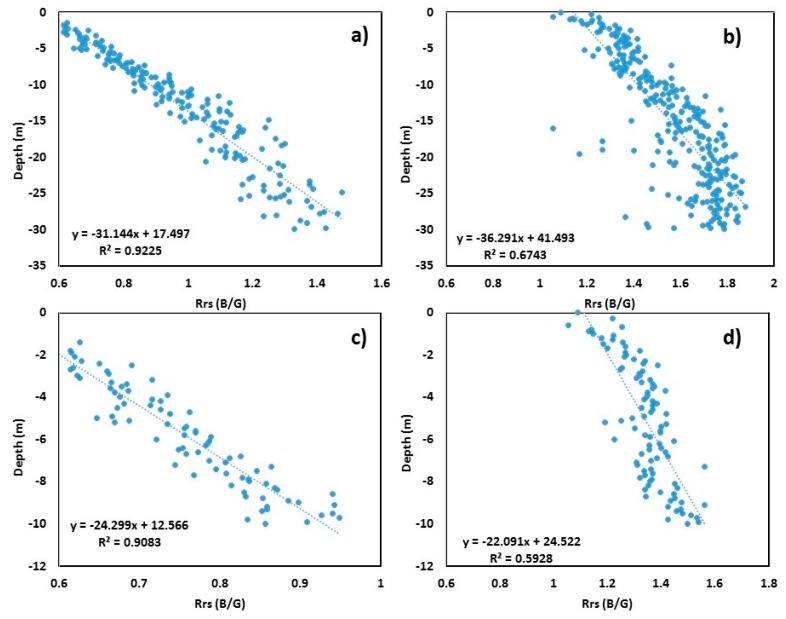
Scatter plots of blue/green (B/G) band versus surveyed depth for Lake Huron: (**a**) Sentinel-2 B/G vs. surveyed bathymetry up to 30 m depth; (**b**) Landsat-8 B/G vs. surveyed bathymetry up to 30 m depth; (**c**) Sentinel-2 B/G vs. surveyed bathymetry up to 10 m depth; (**d**) Landsat-8 B/G vs. surveyed bathymetry up to 10 m depth.

The results obtained from Lake Huron were entirely in contrast with the results from the former two locations. The multiple linear models applied to the 5000 random point values extracted from the B/G band ratioed image and bathymetric DEM are shown in Figure 5. The *R*^2^ values obtained for 30 m depth were 0.92 and 0.67, respectively, for S2 and L8 sensors (*p* < 0.001) (Figure 5a,b), whereas the adjusted depth to 10 m showed a decrease in *R*^2^ value (0.90 and 0.58; *p* < 0.001), as shown in Figure 5c,d. The high value of *R*^2^ between the B/G and bathymetry represented for 30 m clearly indicates the penetration of light into deeper areas in optically clear waters such as lakes.

The equation to derive depth from Sentinel-2 for Lake Huron is as follows:

(5) $$\mathrm{SDB}=-31.14\times \left(\mathrm{Rrs}\;\frac{B}{G}\right)+17.49.$$

The equation to derive depth from Landsat-8 for Lake Huron is as follows:

(6) $$\mathrm{SDB}=-36.29\times \left(\mathrm{Rrs}\;\frac{B}{G}\right)+41.49.$$

### 3.4. Combined Bathymetric Model

**Figure 6:**
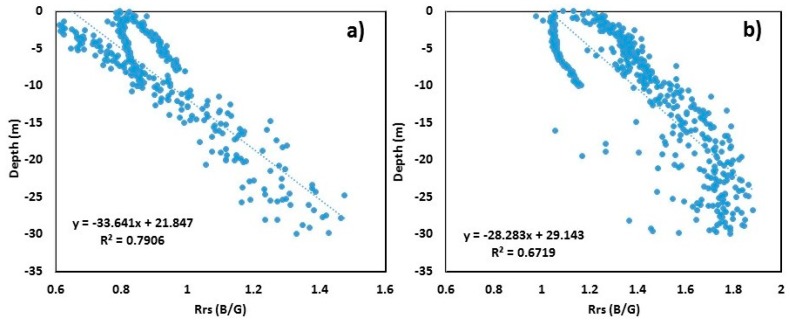
Scatter plots of blue/green (B/G) band versus surveyed depth for all three study areas: (**a**) Sentinel-2 B/G vs. surveyed bathymetry up to 30 m depth; (**b**) Landsat-8 B/G vs. surveyed bathymetry up to 30 m depth.

After obtaining the site-specific bathymetric models, the data from all three study regions were integrated to develop the combined bathymetric algorithm to obtain the regional satellite-derived bathymetry (Figure 6). The *R*^2^ values for the combined bathymetric model (hereafter referred to as an integrated model (IM)) for S2 and L8 were 0.79 and 0.67, respectively (*p* < 0.001).

The equation to derive depth from Sentinel-2 is as follows:

(7) $$\mathrm{SDB}=-33.64\times \left(\mathrm{Rrs}\;\frac{B}{G}\right)+21.84.$$

The equation to derive depth from Landsat-8 is as follows:

(8) $$\mathrm{SDB}=-28.28\times \left(\mathrm{Rrs}\;\frac{B}{G}\right)+29.14.$$

### 3.5. Bathymetric Mapping

**Figure 7:**
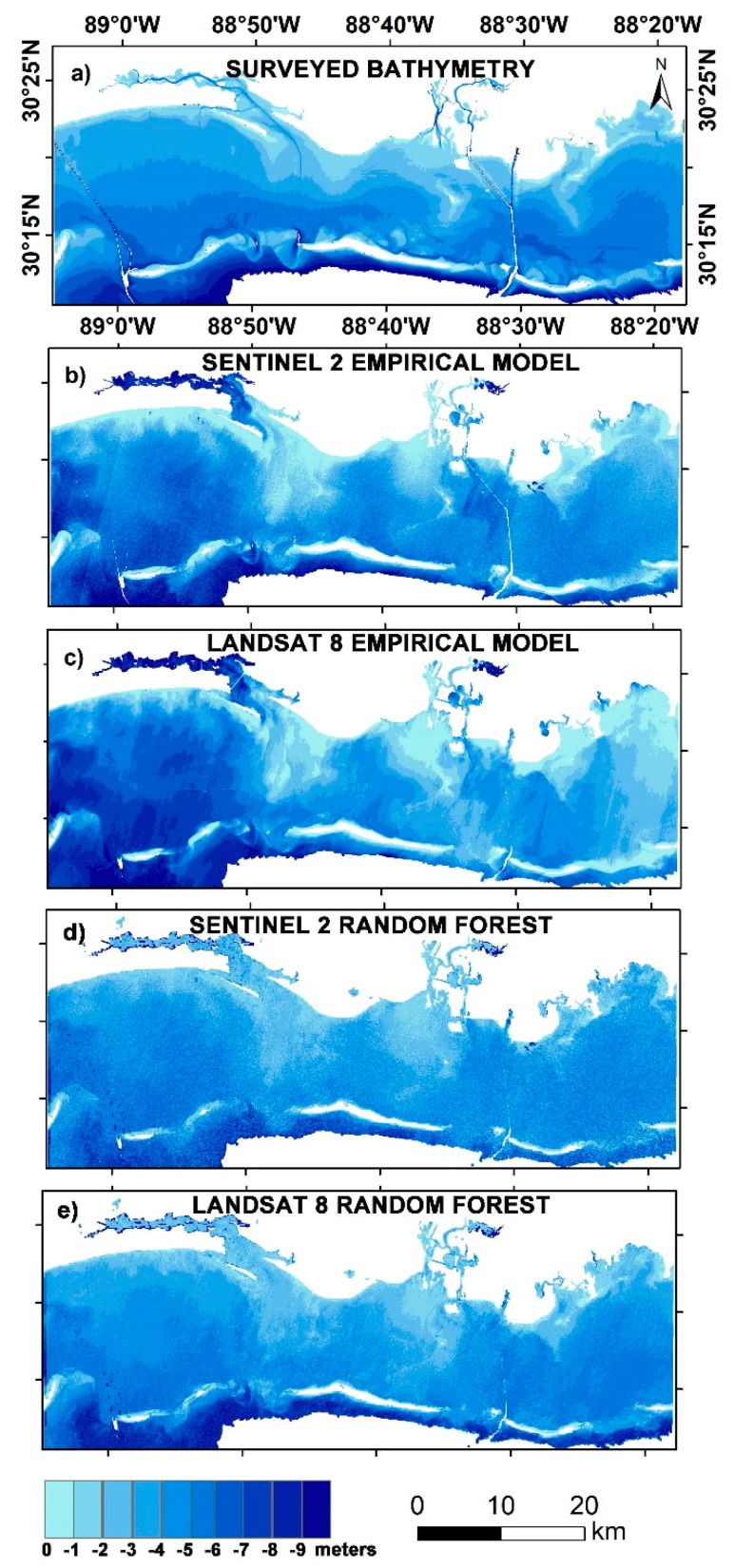
Bathymetric maps of Mobile Bay, Mississippi: (**a**) surveyed bathymetry; (**b**) Sentinel-2 derived bathymetry from empirical approach; (**c**) Landsat-8 derived bathymetry from empirical approach; (**d**) Sentinel-2 derived bathymetry from random forest; (**e**) Landsat-8 derived bathymetry from random forest.

**Figure 8:**
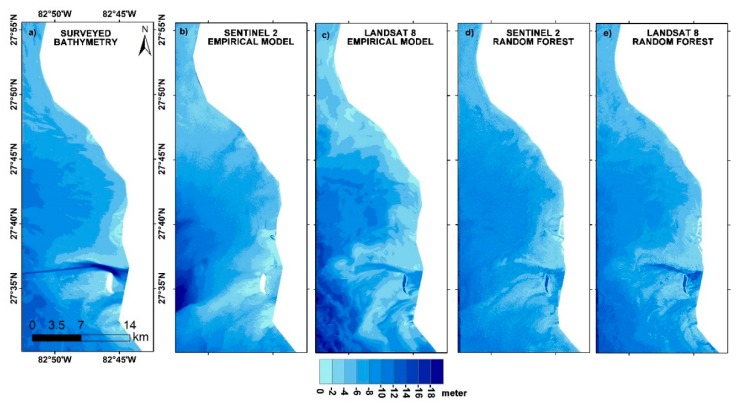
Bathymetric maps of Tampa Bay: (**a**) surveyed bathymetry; (**b**) Sentinel-2 derived bathymetry from empirical approach; (**c**) Landsat-8 derived bathymetry from empirical approach; (**d**) Sentinel-2 derived bathymetry from random forest; (**e**) Landsat-8 derived bathymetry from random forest.

**Figure 9:**
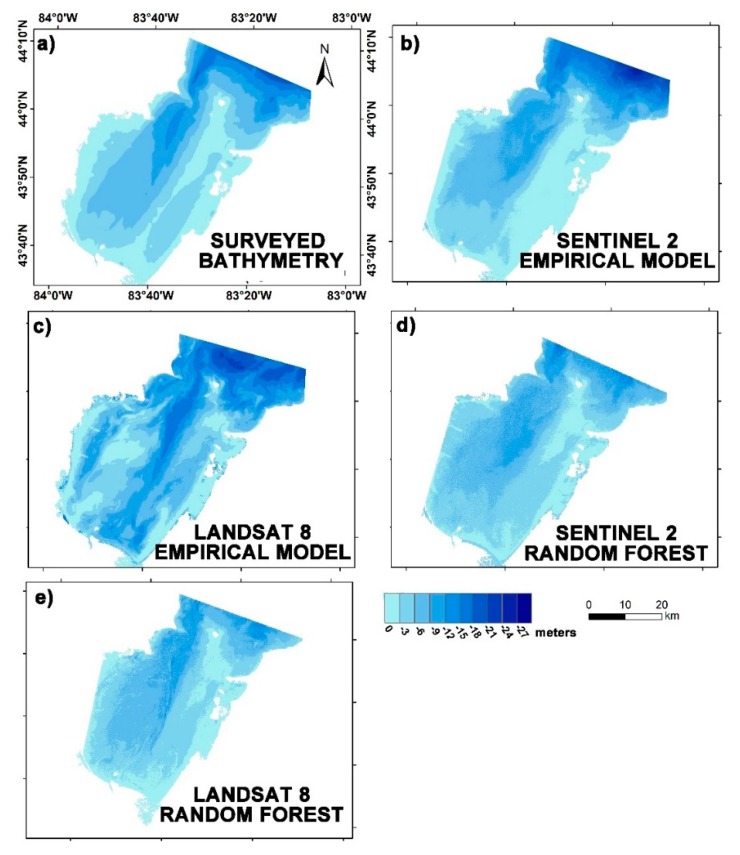
Bathymetric maps of Lake Huron: (**a**) surveyed bathymetry; (**b**) Sentinel-2 derived bathymetry from empirical approach; (**c**) Landsat-8 derived bathymetry from empirical approach; (**d**) Sentinel-2 derived bathymetry from random forest; (**e**) Landsat-8 derived bathymetry from random forest.

Bathymetry for the three study regions was mapped using both the site-specific empirical algorithm and the random forest model. The bathymetric maps derived from S2 and L8 satellite imagery using the empirical approach considering the ratio of blue and green wavelengths, as well as those derived from the random forest model, are shown in Figure 7, Figure 8 and Figure 9. A smoothening filter was applied to the final maps for visual comparison. Given the difference in time of acquisition of bathymetric surveys and that of the satellite image, the direct correlation could not yield a positive result. However, RMSE, MAE, standard deviation (σ), mean, maximum, and minimum values were calculated for the satellite-derived bathymetry for accuracy assessment. Table 2 shows the results of statistical analysis.

**Figure 10:**
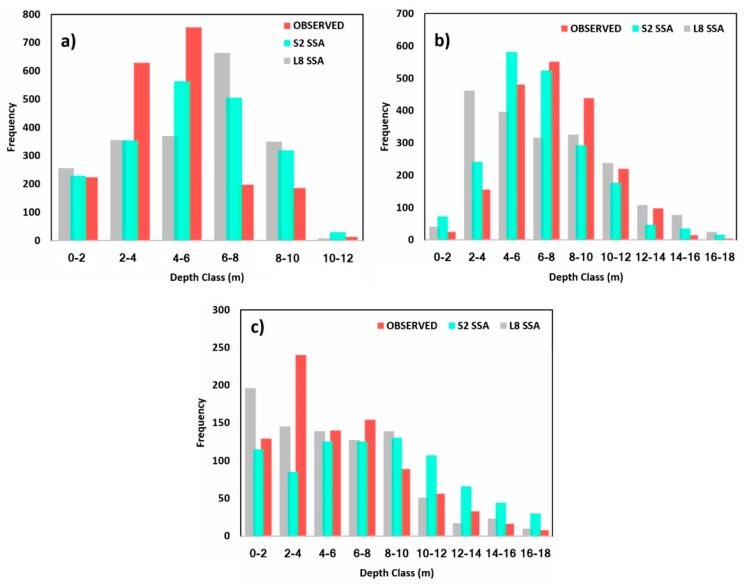
Histogram of depth classes for comparison between different bathymetric maps: (**a**) Mobile Bay; (**b**) Tampa Bay; (**c**) Lake Huron.

The RMSEs estimated from the site-specific empirical algorithm (SSA) for Mobile Bay using 6000 random depth values were 2.26 and 2.54 m, respectively, for S2 and L8 images, whereas the same values for the integrated model (IM) were 4.84 and 5.18 m, respectively (Table 2). Since the RMSEs estimated for the integrated model for both sensors were substantially high (4.84 and 5.18 m), they were not shown on the maps. As observed, significant differences occurred in areas with depths of 2–4 m and 6–8 m (Figure 7a–e, and Figure 10a). Up to 6 m, the S2 SSA algorithm overestimated the depth values and, for depths more than 6 m, the opposite ensued. When using the RF model, the RMSE was considerably improved. For Sentinel data, the estimated RMSE from RF was 1.49 m, whereas, for Landsat data, the RMSE (1.13 m) was even lower than the S2. 

For Tampa Bay, the RMSEs estimated from 3000 random points were 2.80, 2.62, 2.50, and 5.67 m, respectively, for S2 SSA, S2 IM, L8 SSA, and L8 IM. It can be seen from Figure 8a–c and the statistical analysis (Table 2) that L8 SSA-derived bathymetry has the edge over the S2 SSA model. Nevertheless, S2 SSA also produced a good representation of bottom topography (Figure 8b and Figure 10b) with an MAE of only 0.58 m. The random forest model, on the other hand, produced a more accurate SDB (Figure 8d–e), with RMSE values of only 1.95 m (S2) and 1.45 m (L8).

The bathymetric maps produced for Lake Huron using S2 SSA and L8 RF were found to be a close approximation of the actual bottom topography (Figure 9a–e). The RMSEs estimated for S2 SSA and L8 RF were 1.99 and 1.38, respectively. The differences were concentrated in the 0–3 m class, near the southwestern region (Figure 10c). The other models showed RMSEs of 3.30, 4.74, 5.07, and 1.44 m, respectively, for S2 IM, L8 SSA, L8 IM, and S2 RF. Again, because of the larger MAEs and RMSEs, the integrated model results were not shown on the maps.

### 3.6. Bathymetric Profile Analysis

**Figure 11:**
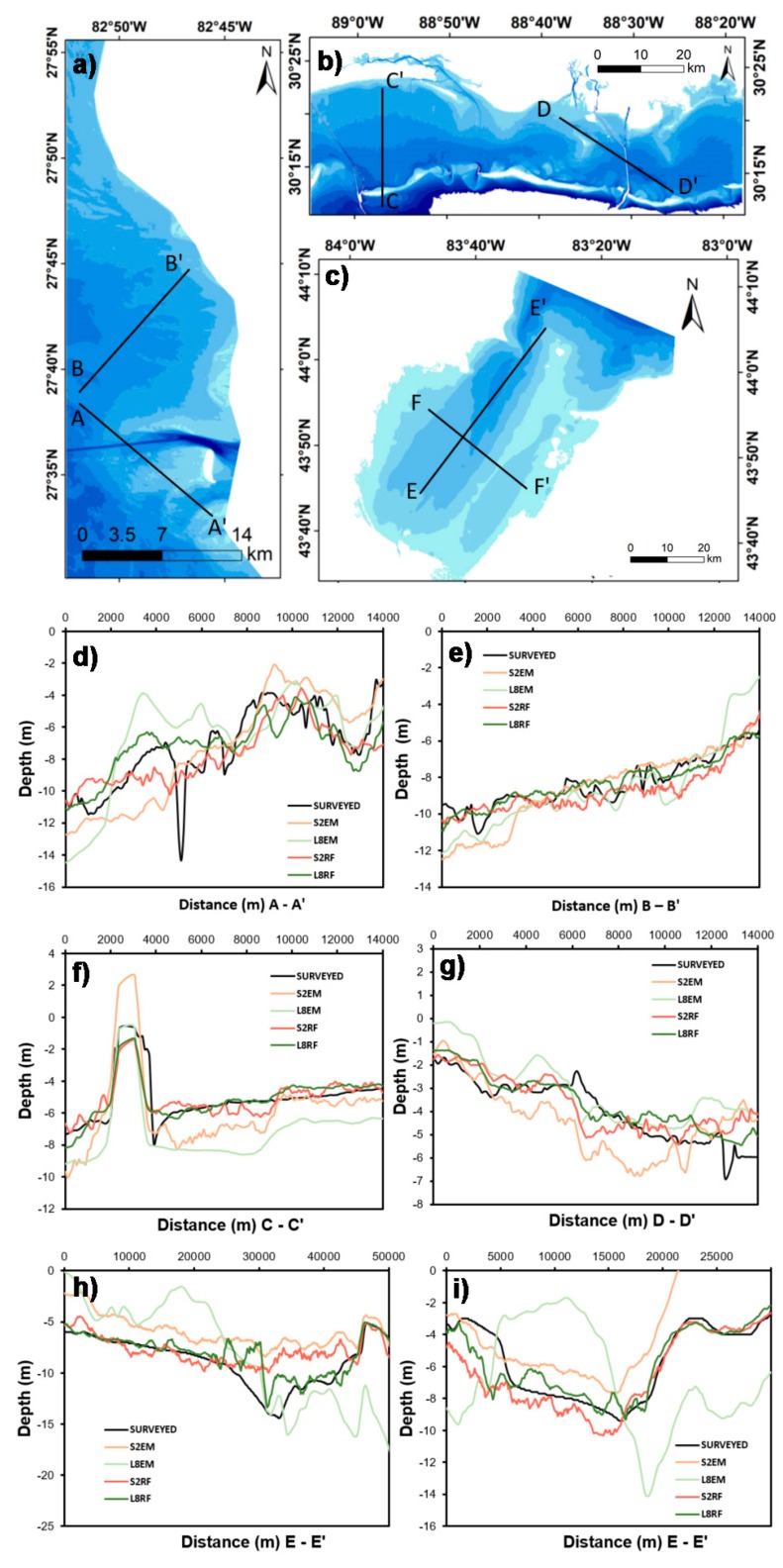
Bathymetric profiles extracted from the empirical and random forest models using Sentinel-2 and Landsat-8 images for Tampa Bay (**a**,**d**,**e**), Mobile Bay (**b**,**f**,**g**), and Lake Huron (**c**,**h**,**i**).

To make a meaningful comparison of the observed bathymetry with the modeled bathymetry, a longitudinal profile analysis was carried out. Because the RMSEs estimated for the integrated model were larger than expected, the profiles were only extracted from the empirically derived bathymetry SSA and the random forest model. For this, using Environmental Systems Research Institute’s (ESRI) ArcGIS, we created line shapefiles for the study region perpendicular to the shoreline. Points along the line were then made at 90-m spacing. Finally, depth values were extracted for each point from the surveyed map, empirically derived bathymetry, and RF-derived bathymetry. Spikes were observed in the S2 SDB because of the higher spatial resolution. Therefore a 9 × 9 window averaging was applied to the S2 derived bathymetry for smoothening the profile. A total of six profiles were extracted for the three study areas (Figure 11).

Figure 11a,d,e report the representative bathymetric profiles for Tampa Bay. Bathymetric profiles derived for Mobile Bay are shown in Figure 11b,f,g, and those for Lake Huron are shown in Figure 11c,h,i. Results show that the RF-derived bathymetric longitudinal profiles exhibited a decent match with the actual profiles (Figure 11). This is a good first-order indication that the RF outperformed the empirical algorithm, and can provide a reasonable estimate of the actual bathymetry. These profiles had at worst 2-m differences in depth. A comparative assessment of S2 and L8 suggests that the L8 RF algorithm-derived profile had the closest match with the actual profile, followed by the S2 RF algorithm and the S2 SSA algorithm. The worst-case scenario was observed for the L8 SSA algorithm-derived profiles for all three study areas. Nevertheless, these six profiles followed the general trend of observed bathymetry.

## 4. Discussion

This study sought to explore the utility of the Sentinel-2 and Landsat-8 multispectral sensors in estimating bathymetric contours across a wide range of aquatic environments. We compared blue–green ratio empirical model findings with those obtained using a data-driven random forest model, to understand the capability of big data for improving the accuracy of satellite-derived bathymetric estimations. This work shows that both Sentinel-2 and Landsat-8 can estimate SDB up to 10 m in coastal waters and up to 30 m in lake waters. As in other earlier related studies, researchers mainly focused on aquatic areas in which there exists not much sediment–water interference for deriving SDB. This is because the depth retrieval in complex waters is limited by water turbidity caused by wave action, suspended sediment, and particulate matter, which limits the penetration of light [7]. However, this study proves that it is still possible to build a predictive model for situations like Mobile Bay and Tamba Bay (Figure 7 and Figure 8), where the environment is more complicated.

The RF method significantly improved bathymetric retrieval when compared to the empirical approach. In particular, the RF results from Landsat-8-retrieved SDB profiles were very well matched with the actual bathymetric profiles (Figure 11). In terms of implementation, the empirical blue–green ratio-based approach has several advantages over the RF method. The ratio model is simple and straightforward and does not require large computation and pre-processing time, unlike its counterpart data-driven models. Moreover, they are descriptive. While data-driven models require a significant amount of pre-processing time, the results are more accurate than regression models [17]. Recently, researchers paid more attention to big data with machine learning for producing accurate bathymetric outputs. Artificial neural networks and support vector machines were successfully used in this regard for estimating the bathymetric depths for different aquatic environments [36,44]. Their analyses also agree with our findings that machine learning models increase bathymetry retrieval accuracy over traditional regression methods.

The increased accuracy of machine learning models compared to the empirical approach can be attributed to the higher number of input data. Furthermore, the accuracy increases sufficiently when providing large training samples [45,46]. Thus, the most porspective way to improve the accuracy of SDB from a machine learning model is to provide image data for all wavelength bands, their band ratios, and log-linear ratios as inputs to the model; however, this is a cumbersome process. Therefore, if the consideration is not solely based on the overall performance but on the balance of overall performance and the computational time, the empirical model is suggested for the first-order indication of bathymetric retrieval. On the other hand, RF models offer improved performance with decent interpretability but require additional computation.

## 5. Conclusions

The bathymetric maps generated by the empirical approach and RF algorithm were by large effective in mapping the bottom topography of Mobile Bay, Tampa Bay, and Lake Huron, despite distinct differences in the morphometry and location. Inherent errors, smoothening, and morphological variation happened for the time differences between the surveyed bathymetry data, and this study was not considered in our analysis. Therefore, it is reasonable to assume that, if outliers were removed, the SSA algorithm and RF model could retrieve depths between 0 and 10 m for coastal areas, and up to 30 m for lake regions in optically clear waters. The random forest model outperformed the empirical algorithms for deriving SDB. Although there was no exact match with the observed profiles, it is clear that, even if the empirical approach is used, the worst-case scenario is a 3-m difference for coastal areas using the Sentinel sensor. To validate the applicability of this method to other regions and for the development of regional bathymetric models, we plan to investigate ways of improving the current model by analyzing more images from different periods, in particular, to examine methods for addressing suspended sediment particles. The results of this study are an excellent indication that both Sentinel-2 and Landsat-8 can be utilized for remotely sensed bathymetry extraction for coastal and lake areas and to complement the data from survey sources.

## Figures and Tables

**Table 1:** Key summary of Landsat-8 Operational Land Imager and Sentinel-2 Multi-Spectral Imager spectral bands, and dates of acquisition, as well as path/row and tile details of images used in this study.

Sentinel-2					Landsat-8			
Band No.	Central Wavelength (nm)		Band Width (nm)	Resolution (m)	Band No.	Central Wavelength (nm)	Band Width (nm)	Resolution (m)
1	443		20	60	1	442	15	30
2	490		65	10	2	482	60	30
3	560		35	10	3	561	57	30
4	665		30	10	4	654	37	30
5	705		15	20	5	864	28	30
6	740		15	20	6	1608	84	30
7	783		20	20	7	2200	186	30
8	842		115	10	8	589	172	15
8b	865		20	20	9	1373	20	30
9	945		20	60	10	1089	59	100
10	1380		30	60	11	1200	101	100
11	1610		90	20				
12	2190		180	20				
Dates of acquisition S2 and L8 scenes								
Mobile Bay		4 January 2016 Tile No. T16RCU			23 April 2016 Path 21 Row 39			
Tampa Bay		14 February 2016 Tile No. T17RLL			20 February 2015 Path 17 Row 41			
Lake Huron		29 June 2016 Tile No. T16TGP			16 April 2016 Path 20 Row 30			

**Table 2:** Statistical analysis of accuracy assessment from different satellite-derived bathymetric models (RMSE—root-mean-square error; MAX—maximum; MIN—minimum; MEAN—average; STD—standard deviation; MAE—mean absolute error; SSA—empirically derived site-specific algorithm; IM—empirically derived integrated model; S2—Sentinel-2; L8—Landsat-8; RF—random forest). All values are in meters (m).

**Mobile Bay**							
	**Empirical Model**					**Random Forest**	
	Surveyed	S2 SSA	S2 IM	L8 SSA	L8 IM	S2 RF	L8 RF
RMSE		2.26	4.84	2.54	5.18	1.49	1.13
MAX	−10.00	−13.32	−14.48	−10. 0	−13.94	−9.55	−9.43
MIN	0.00	3.92	6.25	−0.40	5.45	−0.56	−0.45
MEAN	−4.58	−5.51	−9.21	−5.39	−9.28	−4.42	−4.64
STD	2.20	2.79	1.78	2.71	2.25	1.66	1.89
MAE		0.93	4.63	0.81	4.70	1.10	0.77
**Tampa Bay**							
	**Empirical Model**					**Random Forest**	
	Surveyed	S2 SSA	S2 IM	L8 SSA	L8 IM	S2 RF	L8 RF
RMSE		2.80	2.62	2.50	5.67	1.95	1.45
MAX	−29.63	−32.63	−14.80	−19.87	−7.58	−18.39	−20.83
MIN	0.00	10.36	3.93	4.09	2.68	−0.55	−0.70
MEAN	−7.46	−6.88	−6.37	−7.22	−2.16	−7.43	−7.43
STD	2.82	3.25	1.06	3.73	1.59	2.17	2.37
MAE		0.58	1.09	0.24	5.30	1.25	0.86
**Lake Huron**							
	**Empirical Model**					**Random Forest**	
	Surveyed	S2 SSA	S2 IM	L8 SSA	L8 IM	S2 RF	L8 RF
RMSE		1.99	3.30	4.74	5.07	1.44	1.38
MAX	−18.79	−17.07	−18.68	−22.19	−20.50	−19.38	−15.10
MIN	0.00	9.55	9.87	7.30	5.06	−0.78	−0.10
MEAN	−5.30	−2.61	−4.93	−7.55	−9.38	−5.56	−5.21
STD	3.28	4.25	6.78	4.90	4.25	2.80	2.85
MAE		0.07	2.22	0.79	2.23	0.96	0.93
